# Hydrogen Transportation Behaviour of V–Ni Solid Solution: A First-Principles Investigation

**DOI:** 10.3390/ma14102603

**Published:** 2021-05-17

**Authors:** Jiayao Qin, Zhigao Liu, Wei Zhao, Dianhui Wang, Yanli Zhang, Yan Zhong, Xiaohui Zhang, Zhongmin Wang, Chaohao Hu, Jiangwen Liu

**Affiliations:** 1 Guangxi Key Laboratory of Information Materials, School of Materials Science and Engineering, Guilin University of Electronic Technology, Guilin 541004, China; qjyhsm2012@163.com (J.Q.); liuzhigao0502@163.com (Z.L.); dhwang@guet.edu.cn (D.W.); zhangyanli@guet.edu.cn (Y.Z.); yanzhong@guet.edu.cn (Y.Z.); 2 Key Laboratory of Advanced Energy Storage Materials of Guangdong Province, School of Materials Science and Engineering, South China University of Technology, Guangzhou 510640, China; zhaowei_scut@163.com; 3 College of Materials and Chemical Engineering, Hezhou University, Hezhou 542899, China; zxhui017@163.com

**Keywords:** V–Ni solid solution, hydrogen trapping, H-diffusion properties, tetrahedral interstitial site, first-principles calculations

## Abstract

Hydrogen embrittlement causes deterioration of materials used in metal–hydrogen systems. Alloying is a good option for overcoming this issue. In the present work, first-principles calculations were performed to systematically study the effects of adding Ni on the stability, dissolution, trapping, and diffusion behaviour of interstitial/vacancy H atoms of pure V. The results of lattice dynamics and solution energy analyses showed that the V–Ni solid solutions are dynamically and thermodynamically stable, and adding Ni to pure V can reduce the structural stability of various VH_x_ phases and enhance their resistance to H embrittlement. H atoms preferentially occupy the characteristic tetrahedral interstitial site (TIS) and the octahedral interstitial site (OIS), which are composed by different metal atoms, and rapidly diffuse along both the energetically favourable TIS → TIS and OIS → OIS paths. The trapping energy of monovacancy H atoms revealed that Ni addition could help minimise the H trapping ability of the vacancies and suppress the retention of H in V. Monovacancy defects block the diffusion of H atoms more than the interstitials, as determined from the calculated H-diffusion barrier energy data, whereas Ni doping contributes negligibly toward improving the H-diffusion coefficient.

## 1. Introduction

Hydrogen, which is a clean, efficient, and renewable fuel, is considered as an indispensable energy source for the future owing to the gradual depletion of fossil fuels [1,2]. Hydrogen is also a light atom that is thought to exhibit nuclear quantum effects, which are crucial in describing some transport properties of protons and H atoms [3]. However, to develop hydrogen energy on a large scale, the breakthrough lies in understanding the interactions between hydrogen and materials, such as those related to H-storage and H embrittlement, which can decrease the mechanical properties of metallic materials [4,5,6]. Dense metallic membranes are applied in high-purity hydrogen separation from gaseous mixtures and are likely to form hydrides, especially at high hydrogen partial pressures, thus degrading the mechanical properties of these membranes [7,8].

Vanadium, an ideal hydrogen separation material, has the highest hydrogen permeability, and its cost is lower than the current widely used Pd and its alloys [9,10,11]. Low-activation V has also been identified as one of the most crucial first-wall and blanket material for advanced fusion reactors because it has excellent resistance to neutron irradiation, superior high-temperature mechanical properties, and high compatibility with liquid lithium blankets [12,13,14]. V-based solid solution alloys for hydrogen storage, with a body-centred cubic (bcc) structure, are capable of absorbing/desorbing hydrogen fast at room temperature, have higher capacities, and are crucial for MH/Ni full cell technology. Pure V has a theoretical hydrogen uptake of 3.8% (mass fraction), which is higher than those of the AB5-type (1.4%) and AB-type (1.86%) hydrogen storage alloys. The V-based solid solution alloys are considered as one of the candidate materials for building hydrogen storage tanks for automobile batteries. However, their application as electrode materials in Ni/H batteries is restricted because of their poor electrocatalytic activity and poor discharge capacity; the formation of stable hydrides slows down the dynamic hydrogen absorption/desorption ability of the V-based solid solution alloys, thereby degrading the battery performance. The structure of the alloy changes during the hydrogenation process as follows: bcc(α)→bct(β)→fcc(γ) (where bct is body-centred tetragonal, and fcc is face-centred cubic). Extensive research has been conducted to date to develop feasible solutions for the aforementioned limitation of the vanadium-based solid solution alloys and thus, improve the battery performance [15,16]. Thus, V-based solid solution alloys can be considered as the next-generation materials for vehicle hydrogen storage systems [17,18]. In such applications, the formed stable V hydride is a brittle phase and often becomes the source of fracture under the action of external forces [11]. Additionally, because of the occurrence of substantial vacancy defects, H bubbles are easily formed. This considerably affects the stability of the structural defects in the metal lattice and the mobility of H, resulting in brittle fracture of pure V [13]. H embrittlement is a key problem that restricts the practical applications of V-based solid solution alloys.

Alloying is one of the effective ways to solve this problem, leading to improvements in several properties of these materials [19,20,21]. More specifically, alloying can decrease hydrogen solubility and restrain hydride formation. Experimental studies have shown that hydrogen solubility in transition metal alloys exhibits the following sequence: V–Ti > V–Cr > V–Mn > V–Fe > V–Co > V–Ni [11,14]; Ni doping substantially reduces the hydrogen solubility of pure V, improving its resistance to H embrittlement [22,23,24]. Further, Ni, an effective catalytic component, is widely employed in hydrogen storage materials, chemical fuels, and organic chemical synthesis [25,26]. Moreover, the V–Ni binary alloy system is extensively studied owing to its high hydrogen permeability and mechanical strength [27,28]. Several studies involving density functional theory (DFT) calculations have reported that alloying V with transition metals can decrease its H solubility and embrittlement and enhance the H-diffusion coefficient [27,28,29,30]. However, the vacancy defect mechanism has not been investigated sufficiently. Until now, only a few studies have examined the fundamental mechanism of Ni addition (as a transition metal) and its effect on the interaction between V monovacancies and miscellaneous H atoms. The complex mechanism of the interaction between H and V vacancies has not been elucidated. Therefore, it is crucial to theoretically examine the influence of Ni addition on the stability and diffusion behaviours of interstitial and vacancy H atoms and the trapping of multiple H atoms in the vacancies.

Therefore, to address the abovementioned issues, the formation energies of interstitial H, vacancy H, and H-vacancy clusters were determined using highly accurate first-principles calculations in this study. We comprehensively investigated the stabilities, dissolution, trapping, and diffusion behaviour of H atoms in interstitial positions and vacancies, along with the influence of Ni doping. We expect that the findings of this study will serve as a valuable reference for the industrial development of H-storage, H-separation, reactor first-wall, and blanket systems using V-based alloys, which are promising candidate materials for these applications.

## 2. Materials and Methods

First-principles calculations based on DFT were conducted using the Vienna Ab initio Simulation Package [31,32]. The generalised gradient approximation with the Perdew–Burke–Ernzerhof forms for the exchange-correlation interaction and the projected augmented wave method for the core–valence electron interaction was used [33,34,35]. A 54-atom supercell containing a 3 × 3 × 3 unit cell was used. The V 3d3 4s2, Ni 3d8 4s2, and H 1s1 electrons were regarded as the valence electrons. A kinetic cut-off energy of 360 eV and *k*-meshes with dimensions of 4 × 4 × 4 were applied. While optimising the supercell size, shape, and atomic positions, the convergence threshold for the self-consistency energy was less than 1 × 10^−6^ eV atom^−1^, and the force acting on each atom was less than the maximum of 1 × 10^−2^ eV Å^−1^. The optimum diffusion paths and energy barriers of H atoms between the initial and final configurations were calculated by employing the extremely well-known climbing-image nudged-elastic-band (CI-NEB) method [36]. The phonon spectra and thermodynamic properties were calculated using the PHONOPY code [37].

The solution energy of an interstitial (vacancy) H atom for a metal or alloy is defined as follows [13]:

(1) $$E_{sol}\left(H\right)=E\left(VNiH\right)-E\left(VNi\right)-\frac{1}{2}E\left(H_{2}\right)$$

The vacancy formation energy is calculated as follows [11]:

(2) $$E_{f}\left(vac\right)=E\left(vac\right)-\frac{N-2}{N}E\left(V\right)-E\left(Ni\right)$$

where *E*(*VNiH*), *E*(*VNi*), *E*(*V*), *E*(*Ni*), *E*(*H*_2_), and *E*(*vac*) concretely represent the total energies of the respective systems, and *N* is the number of *V* atoms.

There are two ways to trap H atoms in vacancies, namely trapping them simultaneously and sequentially (one by one). Hence, the trapping energies associated with these two different trapping methods should be defined separately [38]. The average trapping energy of each H atom trapped simultaneously is defined as follows:

(3) $$E_{trap}\left(sim\right)=\frac{1}{n}\left(E_{vac+nH}-E_{vac}\right)-\frac{1}{n}\left(nE_{V,H\left(TIS\right)}-E_{V}\right)$$

The H atoms are sequentially placed in the monovacancy, and the trapping energy of each H atom is given by:

(4) $$E_{trap}\left(seq\right)=\left(E_{vac+nH}-E_{vac+\left(n-1\right)H}\right)-(E_{V,H\left(TIS\right)}-E_{V})\;$$

where *E_vac_*_+*nH*_ and *E_vac_*_+(*n*−1)*H*_ are the total energies of *n* H atoms and (*n* − 1) H atoms in the vacancy of the system, respectively, and *E*_*V*,*H*__(*TIS*)_ refers to the total energy of the H atoms in the tetrahedral interstitial site (*TIS*).

According to the statistical method described by Maroevic et al. [39] and Rao et al. [40], the vacancy concentration can be written as follows:

(5) $$C\left(vac\right)=\sum_{n=0}^{z}\frac{z!}{\left(z-n\right)!n!}{\left(\frac{n_{H}}{\beta N}\right)}^{n}{\left(1-\frac{n_{H}}{\beta N}\right)}^{z-n}\frac{1}{1+\exp\left(\frac{E_{f}+\Delta \varepsilon }{kT}\right)}\;\;\;\;\;\;\;\;\;\;\;\;\;\;\;\;\;\;\;\;\;\;\;\;\;\;\;\;\;\;\;\;\;\;\;\;\;\;\;$$

where *z, n, β, n_H_*/*N, k, T, E_f_,* and *Δε* are the maximum number of trapped H atoms, number of H atoms, number of interstitial sites, H concentration, Boltzmann constant, temperature, vacancy formation energy, and H atom trapping energy, respectively.

## 3. Results and Discussion

**Figure 1:**
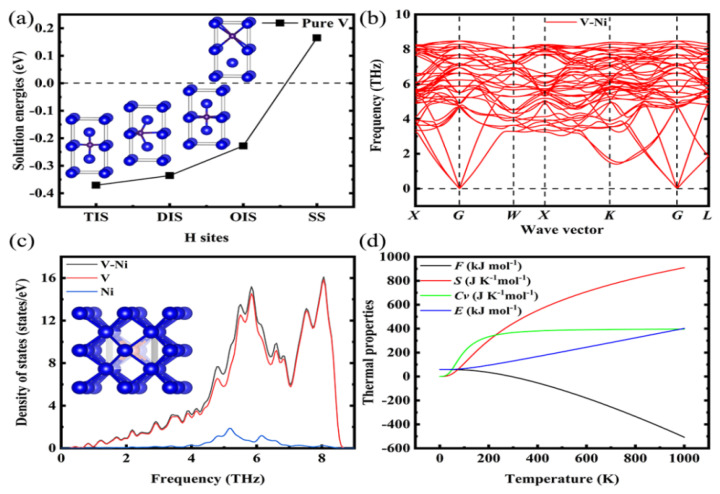
(**a**) Solution energies of H atom in interstitial and substitution sites for pure V; (**b**–**d**) phonon spectra and thermal properties of V–Ni solid solution alloy. The blue, red, and purple spheres represent V, Ni, and H atoms, respectively.

To determine the accuracy of our calculations, the stabilities of the H atoms in V at the (TIS), diagonal interstitial site (DIS), octahedral interstitial site (OIS), and substitution site (SS) were calculated and are plotted in Figure 1a. The solution energies of the H atoms were calculated as −0.371, −0.336, −0.228, and +0.165 eV for the TIS, DIS, OIS, and SS, respectively. Apparently, the most favourable locations for H are the interstitial sites. The experimental H-solution energy is −0.36 ± 0.3 eV [41] for TIS and DIS; the theoretical H-solution energies (after the structural relaxation processes) for TIS, OIS, and SS are −0.374, −0.226, and +1.83 eV, respectively [42]. In addition, our calculations showed that the formation energy of the V vacancy is +2.473 eV, which agrees well with the experimental value of +2.2 ± 0.4 eV [43]. These results prove the validity of the calculation method used in this work.

### 3.1. Structural Stability of V–Ni Solid Solution

To study the influence of Ni doping on the structural stability of metallic V, we built a 2 × 2 × 2 bcc supercell containing 15 V atoms and 1 Ni atom; then the density functional perturbation theory was used to optimise the linear response function and lattice dynamics matrix of the cell to calculate the phonon dispersion and phonon state density. The obtained phonon density of states was used to analyse their thermal properties. As shown in Figure 1b, there are 48 branches in the phonon spectrum, and each branch corresponds to a vibration mode; among these branches, 3 branches with low frequencies correspond to acoustic phonons and 45 branches with high frequencies correspond to optical phonons. The calculated phonon spectrum of the V–Ni solid solution has no imaginary frequency, and thus, the structure exhibits dynamical stability. Figure 1c shows the phonon density of states in the bcc phase of V–Ni, including the total density of states and partial density of states. The peak of the V–Ni solution is at approximately 8 THz, whereas that of Ni is at 5.2 THz. Figure 1d clearly shows the relationship between the thermodynamic properties and temperature. As the temperature increases, the vibrational free energy (*F*) decreases, whereas the entropy (*S*) increases. Moreover, the specific heat at constant volume (*C_v_*) increases rapidly first and then gradually achieves a stable value. These changes were attributed to the lattice thermal vibration and electron heat capacity. In contrast, the internal energy (*E*) of the V–Ni crystals increases significantly with increasing temperatures, and is eventually proportional to the temperature.

**Figure 2:**
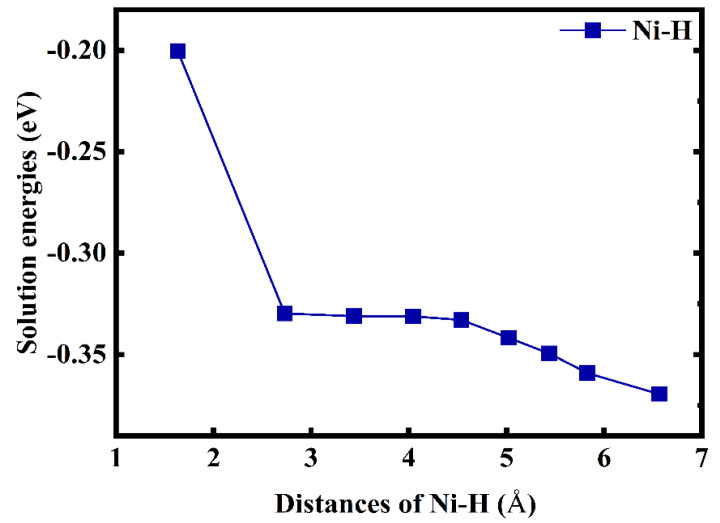
Solution energies of H atom as a function of the Ni–H distances in TIS of V–Ni solid solution alloy.

According to the above results, the H atom at TIS is energetically favourable; thus, it is necessary to further elucidate the interaction between H and Ni atoms. Figure 2 shows the solution energies of H as functions of the Ni–H distances. The distance between the H atom and nearest-neighbour (1NN) Ni atom at TIS is 1.632 Å and the corresponding solution energy is −0.2 eV. This is lower than that (−0.33 eV to −0.37 eV) for the Ni–H distances ranging from 2.728 Å to 6.565 Å, and it is also considerably lower than that in pure V, i.e., −0.371 eV. Interestingly, as the Ni–H distance increased from 1.632 to 2.728 Å, the H-solution energy rised sharply to −0.33 eV. It remained almost unchanged and finally decreased when the Ni–H distance increased from 4.536 Å to 6.565 Å. However, it is worth mentioning that the effect of Ni doping on the solubility of H is highly local, limited largely to within the Ni–H distances. Therefore, Ni doping could significantly weaken the interaction between the V and H atoms and reduce the H-solution energy, thus further improving the resistance of V to H embrittlement.

### 3.2. Stability of H Atoms near Multiple Ni Atoms

**Figure 3:**
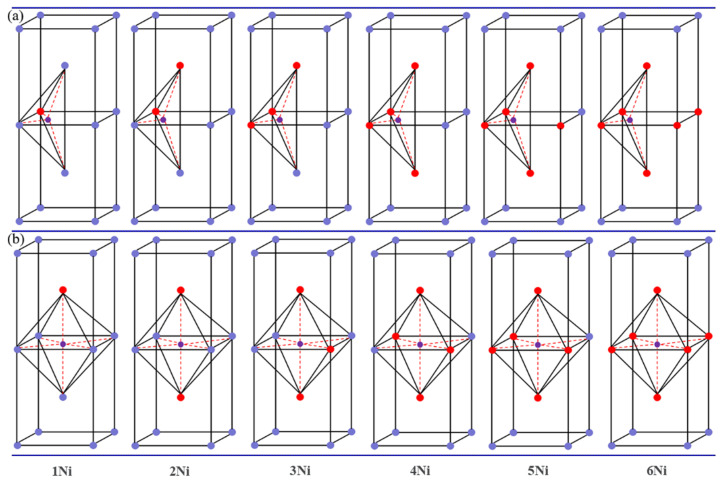
Models showing addition of different numbers of Ni atoms in (**a**) TISs and (**b**) OISs.

**Figure 4:**
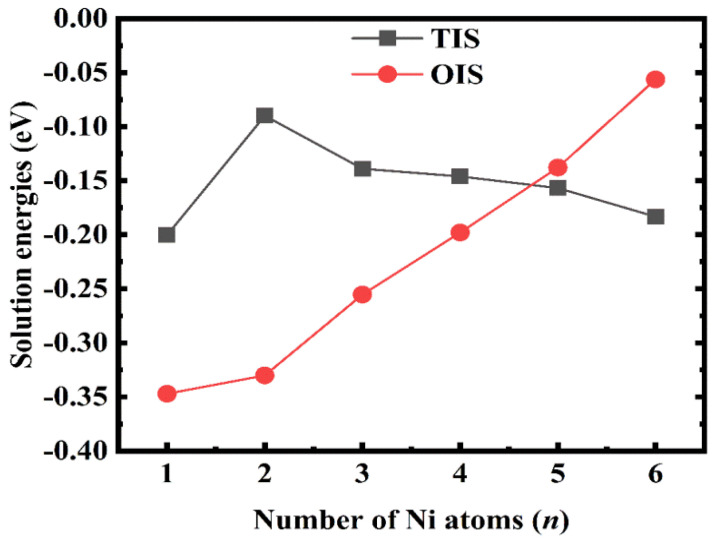
Solution energies of one H atom in TIS and OIS near (*n* = 1–6) Ni atoms.

We further investigated the H-solution properties in TIS and OIS, which are composed of multiple (*n* = 1–6) Ni atoms. The H atom was placed in the TIS and OIS, and Ni atoms replaced its nearest neighbouring V atoms. A series of possible structure models were tested; finally, the most stable configurations were obtained, as shown in Figure 3; the corresponding H-solution energies are summarised in Figure 4. Evidently, the lowest solution energy for H atoms in TIS near two Ni atoms is −0.090 eV, and it gradually increases as the number of nearest Ni atoms increases. The solution energies in all the cases are lower than −0.371 eV, i.e., the H-solution energy in pure V. Meanwhile, the solution energies of H atoms at OIS decreased significantly from −0.347 eV to −0.056 eV, indicating that Ni doping could effectively reduce the stability of the V hydride formed, thus inhibiting H embrittlement of the material.

### 3.3. Interactions between Monovacancies and Multiple H Atoms

**Figure 5:**
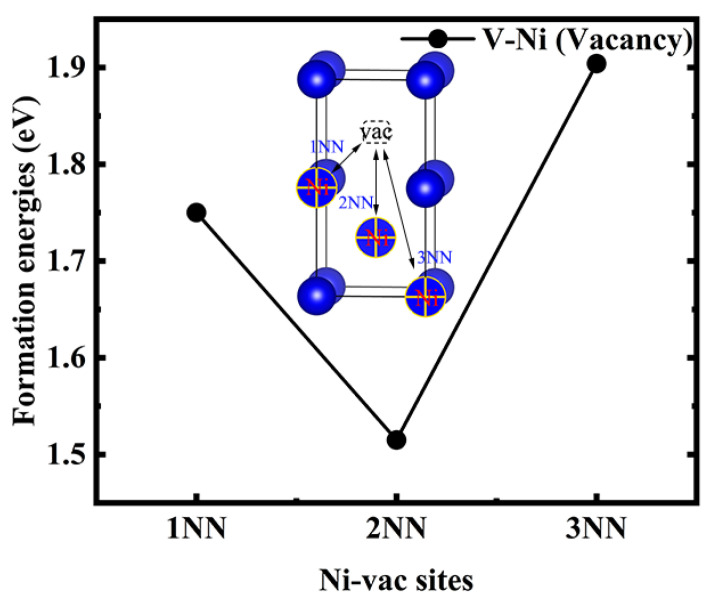
Monovacancy formation energy in V–Ni solid solution alloy.

Vacancy defects are one of the common point defects occurring in metals and alloys. We investigated the influence of introducing Ni, as an additional element, on the stability of the monovacancies in V. By analysing the monovacancy defects around Ni atoms in V, we obtained three structural models, namely 1NN, 2NN, and 3NN sites, using a single vacancy as the reference site, as shown in Figure 5. The formation energy of the 2NN (+1.515 eV) site was lower than those of the 1NN (+1.750 eV) and 3NN sites (+1.904 eV). Thus, the 2NN site in the V–Ni solid solution has the best thermodynamic stability, and it was applied to investigate the interaction between multiple H atoms and a vacancy.

**Figure 6:**
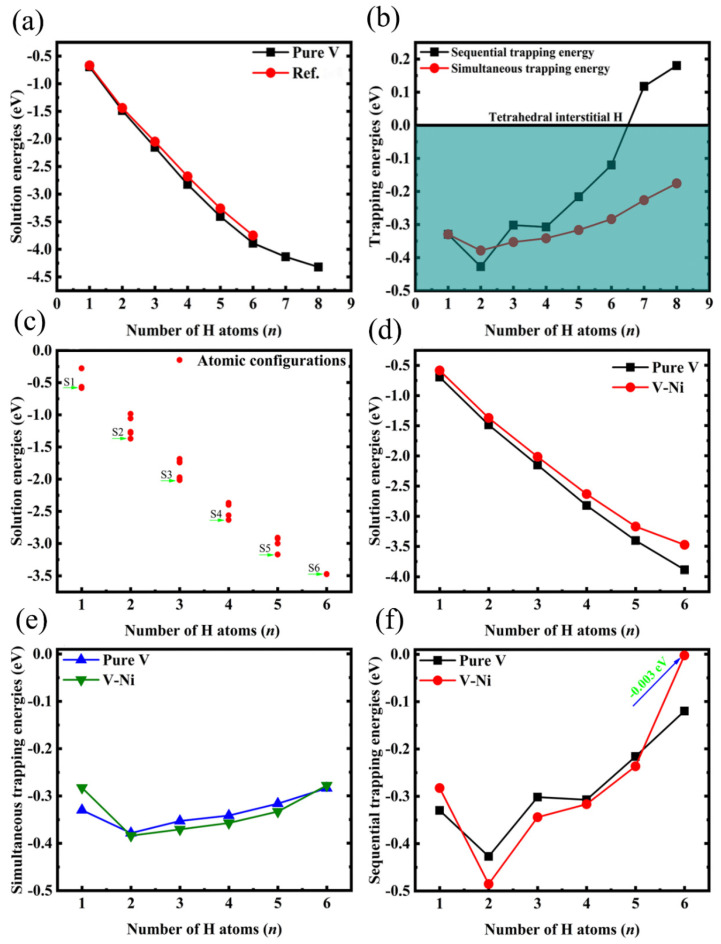
(**a**,**c**,**d**) Solution energies and (**b**,**e**,**f**) trapping energies of multiple H atoms in a monovacancy of V and V–Ni alloy. Reference data are obtained from Pengbo et al. [13].

To investigate the interaction between multiple H atoms and a monovacancy, we first calculated the energetic stability of multiple H atoms at a monovacancy in V. The solution energies of multiple H atoms at the most suitable sites are summarised in Figure 6a. The H atoms in the V monovacancy occupy OIS (with a solution energy of −0.694 eV) instead of TIS and central sites. The corresponding numerical results show excellent agreement with those reported by Pengbo et al. [13]. As the number of H atoms increases, the solution energies gradually continue to increase, indicating that the presence of multiple H atoms at a monovacancy is favourable for forming metal hydrides, thus leading to H embrittlement of the material. Subsequently, the trapping energies of multiple H atoms were calculated, and they are presented in Figure 6b. The sequential trapping energy calculation results indicated that as many as six H atoms were trapped sequentially in a V monovacancy and were separately situated close to six OISs. Moreover, the presence of more than six H atoms is energetically unfavourable, which agrees with the early experimental results reported by Myers [44]. Six H atoms can be trapped at the nearest neighbouring OIS in a bcc metal monovacancy. The calculated simultaneous trapping energy results showed that at least eight H atoms could be trapped, and the H atom in the stable OIS started to shift toward the TIS when the number of H atoms exceeded six. However, Gui et al. [38] reported that a monovacancy in V can trap a maximum of 12 H atoms simultaneously based on first-principles calculations.

We calculated all the possible configurations involving multiple H atoms when an Ni atom is added. As shown in Figure 6c, the corresponding solution energies increase gradually as the number of H atoms increases, indicating that the stability of H in the vacancy is stronger and the solubility of H involves an exothermic process. For comparison, the solution energies of multiple H atoms in the bulk V monovacancy before and after Ni addition are presented in Figure 6d. After adding Ni, the stability of V hydrides decreased, thereby inhibiting their H embrittlement. Figure 6e,f present the trapping energies as functions of the H atom number. Obviously, the trapping energies of two–five H atoms sequentially or simultaneously trapped in the V–Ni monovacancy are higher than those of the corresponding V monovacancy. However, the trapped six H atoms exhibited very poor affinity toward the monovacancy, with simultaneous trapping energies of −0.003 eV, thereby indicating effective suppression of the H retention. Therefore, from the perspective of the trapping energy, the V–Ni monovacancy can capture up to six H atoms.

### 3.4. Monovacancy and Vac-nH Cluster Concentrations

**Figure 7:**
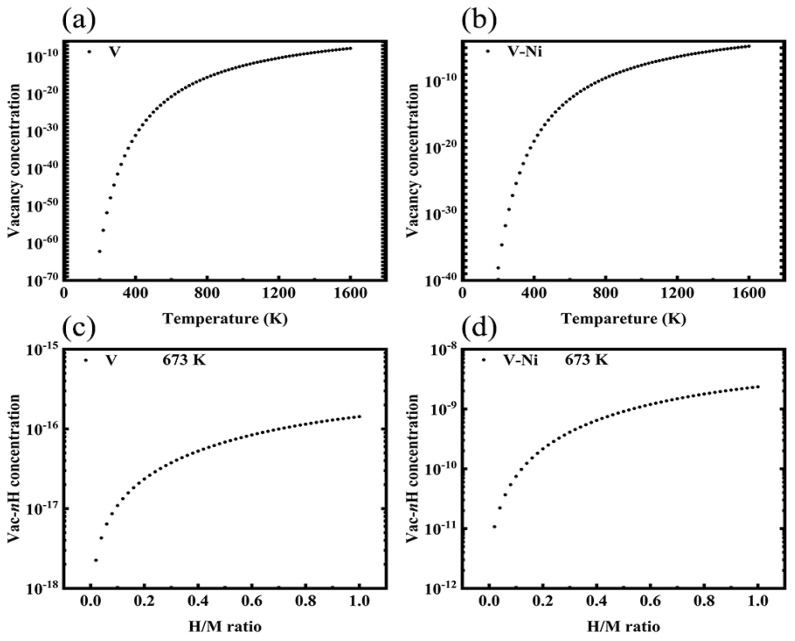
(**a**,**b**) Monovacancy concentration as a function of temperature; (**c**,**d**) functional relationship between Vac-*n*H cluster and H/M ratio.

To study the equilibrium concentrations of vacancies, we employed a statistical method [39,40] to estimate the intrinsic vacancy and Vac-*n*H cluster concentrations. The calculation results are presented in Figure 7. As observed in Figure 7a,b, the concentrations of V and V–Ni vacancies gradually increase with the increase in temperature, which means that the vacancy concentration can easily damage metals or alloys at high temperatures. As for the Vac-*n*H cluster concentration shown in Figure 7c,d, when the H/M ratio (H concentration) gradually increases at an operating temperature of 673 K, the Vac-*n*H cluster concentration also increases accordingly, thus proving that a high Vac-*n*H cluster concentration leads to H embrittlement. This finding is consistent with the observations of Matsumoto [45], who proved experimentally that sample cracking occurred when the H concentration in a V-based alloy exceeded *C* (H/M) = 0.2. The concentrations of the monovacancy and Vac-*n*H cluster in Ni-doped V increased rapidly; however, the concentrations of V and V–Ni monovacancies were 7.675 × 10^−32^ and 8.679 × 10^−20^ at 400 K, respectively.

### 3.5. H Diffusion in Interstitials and Vacancies

**Figure 8:**
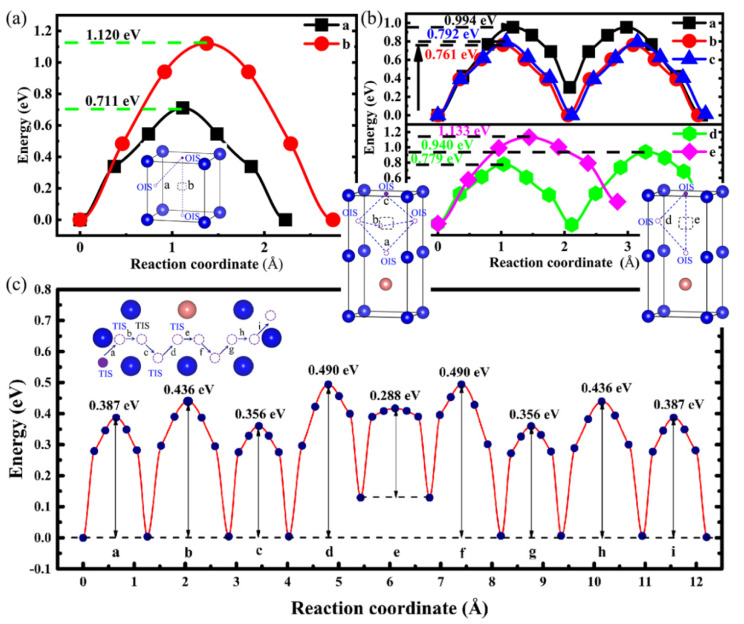
Diffusion paths and energy barrier for H atom in (**a**) V, (**b**) V–Ni vacancy, and (**c**) V–Ni interstitial.

To further study the influence of Ni addition on the H atom diffusion in interstitials and vacancies, we calculated the possible diffusion paths and migration energy barriers of H diffusion using the CI-NEB method [36], as shown in Figure 8. However, we only considered the H atom diffusion between the most stable absorption sites. Based on our previous work [46], the diffusion energy barriers of H in perfect bulk V from TIS to TIS and TIS to OIS are 0.132 and 0.20 eV, respectively, indicating that the optimal diffusion paths are located between TISs (TIS → TIS). The calculated energy barriers for H are 0.711 and 1.120 eV from OIS to OIS and from OIS to the vacancy centre, respectively, when a monovacancy defect occurs in pure V, as seen in Figure 8a, suggesting that the H atom in the vacancy easily diffuses along the OIS → OIS path with the most favourable energetic state. Compared with the vacancy diffusion energy barriers, the H atom prefers migrating to interstitials because it only needs to overcome a small energy barrier.

After adding Ni atoms, as illustrated in Figure 8b, the H atom in the vacancy migrated from one OIS to another nearest neighbouring (1NN) OIS through various possible diffusion paths, with each path passing through an intermediate transition state. The computed energy barriers along paths a, b, c, d, and e were 0.994, 0.761, 0.792, 0.940, and 1.133 eV, respectively; thus, the optimal path for H diffusion is path b. Furthermore, we simulated the diffusion behaviour of interstitial H, as depicted in Figure 8c. The H atom jumps from the first TIS on the left to the last TIS and moves closer to the Ni atoms in the middle with a low energy barrier of 0.288 eV, whereas the other paths have extremely high energy barriers, especially paths b and d. From a kinetic perspective, when the H atom gradually migrates to the TIS near the Ni atom (a → b → c → d → e: 5NN TIS diffusing to 1NN TIS), the H migration barrier tends to first increase and then decrease. The addition of Ni atoms does not reduce the H-diffusion energy barrier compared with that observed in pure V; this could be attributed to the elastic effects and electronic properties of V–Ni [27]. In addition, the H atom constrained in the inner cavity of the vacancy needs to overcome the high energy barrier to escape from it. Therefore, the presence of Ni could control the nucleation and blistering of H in V.

## 4. Conclusions

We investigated the hydrogen transportation behaviour of Ni-doped vanadium solid solution using first-principles DFT calculations. The calculation results showed that the monovacancy formation energies of V and the V–Ni solid solution alloy were +2.473 and +1.515 eV, respectively. Single H atoms in interstitials and vacancies preferred to occupy the TIS and OIS, respectively, owing to their low solution energy, and diffused along the TIS → TIS and OIS → OIS paths with low energy barriers. H diffusion preferentially occurred in the interstitial sites rather than in the vacancies that were stronger traps for H atoms. Ni doping could reduce H solubility, inhibit H retention, and provide more diffusion paths. The monovacancy and Vac-*n*H cluster concentrations depend on the temperature and H concentration over a wide range. This study clarifies the interactions between H atoms and vacancy defects. This understanding provides insights into the trapping of multiple H atoms or the formation of H bubbles during H permeation, thus offering a theoretical foundation for designing alloy membrane materials.

## Data Availability

The data presented in this study are available on request from the corresponding author.
